# Benefits of promoting scholarship among program directors: promoting scholarship among directors is a win-win-win for institutions, trainees, and directors

**DOI:** 10.3389/frma.2024.1477471

**Published:** 2024-11-20

**Authors:** Patrick D. Brandt, Donita L. Robinson

**Affiliations:** ^1^Office of Graduate Education, School of Medicine, University of North Carolina at Chapel Hill, Chapel Hill, NC, United States; ^2^Department of Psychiatry and Bowles Center for Alcohol Studies, School of Medicine, University of North Carolina at Chapel Hill, Chapel Hill, NC, United States

**Keywords:** program directors, scholarship, program evaluation, higher education, life science training

## Abstract

When the National Institutes of Health (NIH) budget doubled in the late 1990s, it led to a rise in the number of PhD-trained scientists and to increased NIH-funded programs to diversify the biomedical workforce. This trend has seen more PhD scientists take on leadership roles as program directors in academia. These program directors are often highly skilled in research design and data analysis, and they bring a scholarly approach to their administrative duties. Despite organizational challenges, promoting scholarship among program directors offers numerous benefits, including enhanced institutional reputation and better training outcomes. Herein we use examples from peer reviewed literature to illustrate how publications by program directors have influenced national policies and practices in biomedical training. Encouraging more academic institutions to support program director scholarship can yield significant returns for institutions, trainees, and the directors themselves.

## Introduction

The doubling of the National Institutes of Health (NIH) budget in the late 1990s was followed by an increase in the number of PhD-trained scientists and by NIH-funded programs aimed at diversifying the biomedical workforce and supporting trainees at all levels. These programs have been increasingly led by program directors with PhD experience, reflecting the broader trend of integrating highly trained scientists into leadership roles. For instance, the Graduate Career Consortium, a professional association dedicated to the career and professional development of graduate and postdoctoral scholars, has seen its membership grow from a few dozen in the 1990s to more than 500 in 2022. Notably, 60% of these members have followed a career path involving PhD training (Annual Member Survey, 2020).

Program directors who have earned a PhD are trained to analyze complex systems, pose testable hypotheses, publish their findings, and keep up with quickly evolving fields by immersing themselves in the relevant peer reviewed literature. A benefit of this growing community of research-ready program directors is that they approach higher education administration similarly to how they managed their research projects. That is, with curiosity and an eye on publishing peer-reviewed articles on best practices, sharing results of interventional strategies, and publishing professional outcomes of large populations of trainees. Indeed, this is a growing literature: Van Wart et al. (2023) have characterized the emerging field of biomedical training publications since 1950 and reported that after remaining largely flat for four decades, articles related to evidence-based studies in research training, combined with descriptive programmatic articles, tripled in the 1990s, then quadrupled in the 2000s, and then more than doubled in the 2010s compared in each case to the decade before (Van Wart et al., 2023).

Yet, the organizational structure and job responsibilities of many PhD-trained program directors do not allow them to dedicate a portion of their time to scholarship. Like many academic faculty and professionals, program directors tend to be stretched thin, spending most or all their effort to run programs with little time for scholarly pursuits. Resisting this tendency, we assert in this article that promoting scholarship among directors is a win-win-win for institutions, their trainees, and the directors themselves.

We are part of the Office of Graduate Education in the School of Medicine at the University of North Carolina at Chapel, which has hired more than a dozen PhD-trained program directors to lead diversity initiatives, first-year graduate training programs, and career and professional development programs over the past 15 years. In our division, director positions are designated by Human Resources policies as staff rather than faculty positions, while across campus in the UNC Graduate School, positions with similar scope and responsibility are classified as faculty. This matters because faculty positions, regardless of whether they are tenure-track, typically have an expectation of scholarship (whether or not they have protected time to do so), while non-faculty positions do not. Nevertheless, we have found in our office that promoting scholarship by carving out time for research and writing in director-level positions has returned numerous benefits to the institution, the trainees, and the directors—some of which may not be obvious from the outset. We will illustrate this point with a few prominent examples of peer-reviewed publications that gained national importance and have impacted biomedical training and national policy. It is outside of the scope of this perspective to do an exhaustive review of all the impactful and important contributions in this field, but those presented are illustrative of the benefits. By enumerating these benefits, we hope to encourage more academic institutions to promote scholarship and publication by program directors, regardless of their faculty status.

## Data-driven predictors of success in biomedical training programs

Graduate and postdoctoral training programs collect large amounts of data related to admissions, benchmarks, and outcomes. National initiatives and annual conferences serve as collaboration incubators where program directors, faculty deans, and funders discuss intervention ideas and needed policy changes. The following groups promote many of these spaces where collaborators organize and publications are envisioned:

- NIH-funded broadening experiences in scientific training grants (Lara et al., 2020).
- Next generation life sciences coalition (https://nglscoalition.org/).
- Annual meetings of the NIH Training and Workforce Development programs.
- Annual meetings of the American Association of Medical Colleges (AAMC) Graduate Research Education and Training (GREAT) Group.
- Annual meetings of the Graduate Career Consortium.

Program directors are accustomed to attending scientific meetings to hear the latest advances and brainstorm collaborations with colleagues new and old. The following paragraphs discuss examples of scholarship that have resulted from this process.

In 2017, two studies were published in tandem that evaluated which aspects of graduate student applications predict success in dissertation programs, with a specific interest in the Graduate Record Exam (GRE). One paper was from Moneta-Koehler et al. (2017) at Vanderbilt University, and the other was from Hall et al. (2017) in our office. Both papers analyzed enrollment data from hundreds of students matriculating into biomedical umbrella programs over several years to reveal that GRE scores were unrelated to metrics of doctoral student success, such as completing the PhD, time to degree, and number of papers published. These papers provided data that allowed biomedical doctoral programs to reconsider how much weight to give GRE scores in admissions selection or requiring GRE scores at all. As a result, these papers contributed to the discontinuation of a GRE requirement at over 400 PhD programs (colloquially coined “GRExit”) as well as several follow-up studies (e.g., Petersen et al., 2018; Walters et al., 2022; Bridgeman and Cline, 2022; Williams et al., 2021). This decision impacted both applicants who no longer needed to pay for expensive exams and preparatory courses, and admissions committees who often saw increased number or diversity of applicants. Moreover, one of the authors (Dr. J.D. Hall) compiled a list of biomedical doctoral programs that did not require the GRE, a list that he still maintains (see list at https://docs.google.com/spreadsheets/d/1MYcxZMhf97H5Uxr2Y7XndHn6eEC5oO8XWQi2PU5jLxQ/edit#gid=0). The publication and the public list of doctoral programs established Dr. Hall as an expert in the utility of the GRE in biomedical doctoral admissions, and he was asked to give presentations at conferences and at universities that were deliberating on whether to continue GRE requirements (Dr. J.D. Hall, personal communication).

Another example of valuable dissemination of evidence-based information comes from a 2021 publication by Brandt et al. (2021) that presented a cross-institutional analysis of the effects of trainee professional development on research productivity. This study addressed the question of whether increasing participation in career and professional development programming leads to lower research productivity compared to trainees who participate in fewer career and professional development activities. Professional development participation data was collected from 1,750 trainees across 10 institutions during a 4-year period. The data provided no evidence that participation in career and professional development, even at the highest amounts, increased time to degree or decreased publication productivity. This large-scale study gives trainee advocates (such as funders), graduate student and postdoctoral offices, university leaders, and individual faculty advisors data they can use to promote and fund professional development initiatives without concern for unintended negative consequences on student success.

## Programmatic best practices

In addition to evidence-based studies, program directors contribute in a scholarly way by publishing best practices and descriptive programmatic articles. These articles may have a niche readership and unremarkable citation metrics, but they often have an outsized impact because they provide the recipe for replicating a successful program at a new institution.

An example of a programmatic paper from our office is Hall et al. (2016) describing a 12-month biomedical post-baccalaureate training program. In its goal to increase diversity in the biomedical scientific workforce, NIH aimed to support scientists in the transition from undergraduate to graduate school via the Post-baccalaureate Research Education Program (PREP) R25 grant mechanism, introduced in 2003 and expanded in recent years. When developing the framework for PREP at the University of North Carolina (UNC PREP), the program directors incorporated literature indicating the need to cultivate a “scientific identity” and affirm to trainees that they indeed belonged in the scientific community (Hurtado et al., 2009; Gazley et al., 2014; Gibbs et al., 2014; Carlone and Johnson, 2007). After 5 years of program outcomes were collected, Hall et al. published outcome data along with a framework for program design, with a focus on scientific identity promoted in part by having PhD-trained scientists running the program. In the intervening years, as the NIH PREP funding mechanism has grown, various institutions have used that publication as a guide when crafting new applications and post-baccalaureate programs—in this way, UNC PREP has emerged as a de facto exemplar and a leading PREP program. Recently, extended outcomes of several NIH-funded PREP programs in the mid-Atlantic region were compiled and published, disseminating a yet broader compilation of best practices (Wright et al., 2024).

In another example, Santo Domingo et al. (2019) published on the success of the 30-year-old Meyerhoff Program at the University of Maryland Baltimore County. The Meyerhoff Program offers financial assistance, mentoring, advising, and research experience to diverse undergraduate students committed to obtaining Ph.D. degrees in math, science, and engineering. The publication contained details about how the program can be adapted and adopted at other institutions. Examples of two other institutions in the beginning stages of implementation were shared in the article, including the University of North Carolina's Chancellors STEM Scholars and Pennsylvania State University's Millenium Scholars Program. Soon after the publication, other programs were created based on the Meyerhoff model including the SEED Scholars program at Berkeley, the Karsh STEM Scholars at Howard, and the PATHS Scholars at UC San Diego. Other existing and emerging programs no doubt borrowed elements from the Meyerhoff model, thanks to the effort put in to prepare and disseminate the manuscript (Santo Domingo et al., 2019).

A third example centers around professional internships for trainees in the biomedical sciences. Prior to the mid-2010s, internship opportunities for PhD candidates in the life sciences were rare, but that is changing now. There are many unique aspects of the graduate training apprenticeship and funding model that create barriers to internships and other types of off-site experiential skill acquisition. Some programs are finding ways around those obstacles and publishing their methods and outcomes. In 2018, Schnoes et al. were among the first to publish details of a successful internship program for graduate students, specifically at the University of California, San Fransisco. The UCSF internship model was replicated at the University of California, Davis with equal success, and the manuscript shares details of how the program can be adapted at other sites (Schnoes et al., 2018). Another peer-reviewed, outcome-focused, internship program overview was published by our office in 2023 to share a successful template for a graduate student internship program (Brandt et al., 2023). A mixed-methods approach was used to evaluate the success of the first 5 years of the internship program. Benefits to interns, research advisors, and host companies were described; research advisor support for the program was quantified over time; and the discussion addressed lessons learned, persistent challenges, and advice for implementation at other institutions (Brandt et al., 2023).

Descriptions of smaller scale interventions are also worth publishing. For such articles to be accepted in peer-reviewed journals, there is usually a rigorous qualitative or quantitative analysis of outcomes that accompanies the program description. A few notable publications in this vein include the description of a business management course for scientists at Vanderbilt University (Petrie et al., 2017); a graduate level career development course at Yale (Claydon et al., 2021); and a cross-institutional industry site visit program in the Research Triangle region of North Carolina (Collins et al., 2020).

## Benefits of institutional investment in scholarship

Dissemination of higher education research and best practices carries many benefits to the broader biomedical training enterprise, but what about the specific institution supporting that scholarship? Data collection, analysis and writing can take time away from running the programs for which directors are hired. Nevertheless, we think this exchange is worthwhile due to a range of direct benefits to the supporting institution.

Key among these benefits is that having an eye on scholarship and program evaluation helps to secure and maintain grant funding. Federally sponsored programs such as NIH-funded institutional training programs benefit enormously from evidence-based training plans. Moreover, the fact that much of the research we cite in our training proposals was generated at UNC establishes our community as experts, showcasing both the training environment and the institution's commitment. This promotes a virtuous cycle of institutional support, funding success, strong programs with favorable outcomes, and increased national reputation; all of which build upon each other.

There are significant benefits that come to program directors themselves when they are encouraged to publish in peer reviewed journals. Publishing and keeping abreast of evidence-based best practices can build connections and expand networks in a way that makes work more meaningful and effective. There is also synergy between publishing data that impacts national policy and emerging as a leader in the field, such as Dr. Hall's experience after publishing on the GRE as a predictor of student success in biomedical doctoral programs.

## Suggestions for promoting scholarship by program directors

We offer the following recommendations to institutions so that they and their program directors can realize the benefits listed above.

1) As programs are envisioned and initiated, proceed with a plan to evaluate the results of the intervention at different time points. The data that result from that evaluation can often be published and will likely be valuable to other universities. This is especially true when there is a multi-site intervention such as was the case with the NIH Broadening Experiences in Scientific Training grants. Be sure to ask your institutional review board whether you need human-subject research approval prior to publication.
2) Include scholarship as a protected effort in new position descriptions. It may not be feasible for current program directors to take on more responsibilities without considering what scholarship will synergize with current duties and what duties can be replaced by it. The intent is not to add yet another responsibility to a program director's already full plate, but to find ways to free up their time for scholarship in light of the benefits to the institution and its educational mission. A conversation between program directors and supervisors is important because not all program directors may want to develop in this area.
3) Finally, celebrating scholarship achievements in the annual review process and showcasing them within the institutional and community will reward the time spent to disseminate information beyond the university.

Research institutions invest heavily in biomedical doctoral training, and many sectors of the economy benefit from the influx of highly trained and scientifically minded PhD trainees, including the pharmaceutical research and development industry, the clinical trials enterprise, and the business development and entrepreneurial sectors. Of course, many PhD graduates also apply their training in academia as faculty, program directors, administrators, and deans. As many program directors come to their position from a research background, they view themselves as scientists and value scholarly output as a measure of success and national reputation. In this way, having protected time for scholarship is motivating and improves retention of valuable employees. By enumerating these benefits here, we hope to encourage more academic institutions to promote scholarship and publication by program directors, whether or not they hold faculty positions.

## Data Availability

The original contributions presented in the study are included in the article/supplementary material, further inquiries can be directed to the corresponding author.
